# Supplementary material for: Individual Differences in Multisensory Interactions: The Influence of Temporal Phase Coherence and Auditory Salience on Visual Contrast Sensitivity
Source: Vision (Basel). 2020 Feb 5;4(1):12. doi: 10.3390/vision4010012 (PMC7157667; doi:10.3390/vision4010012)
Supplement: Supplementary file 1 [file vision-04-00012-s001.pdf]

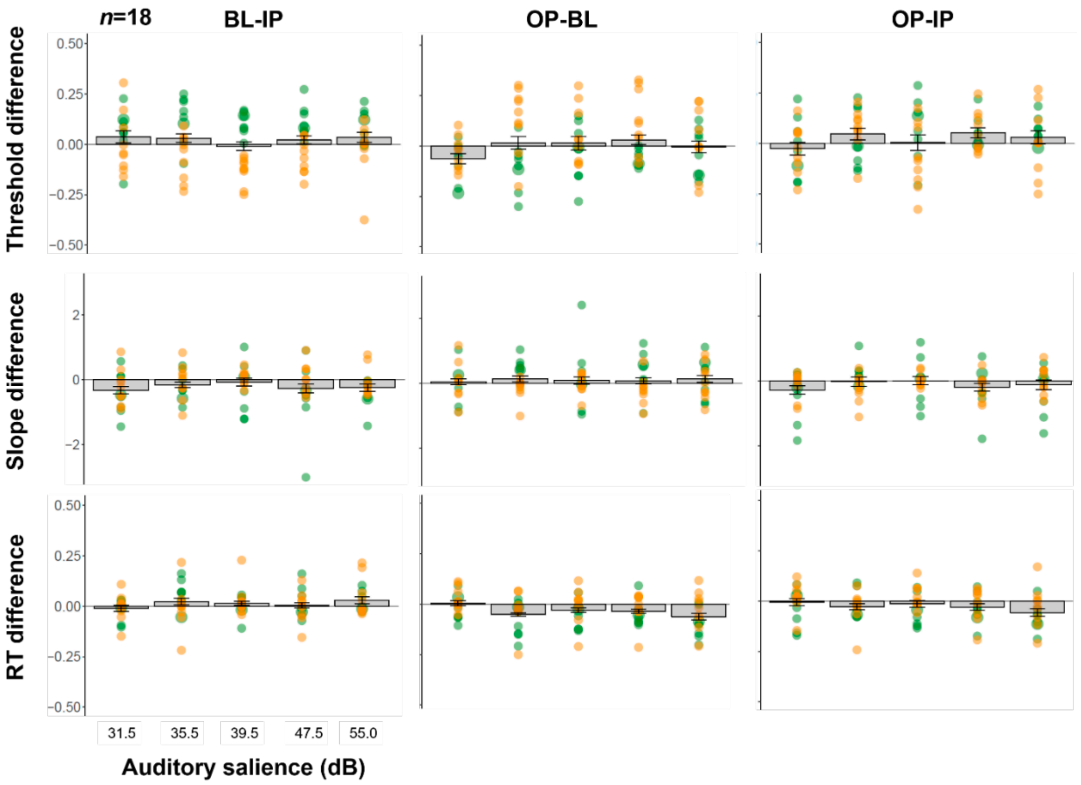

Figure S1. Threshold, slope, and RT difference across auditory salience levels. Green: Enhancement group; Orange: Suppression group.

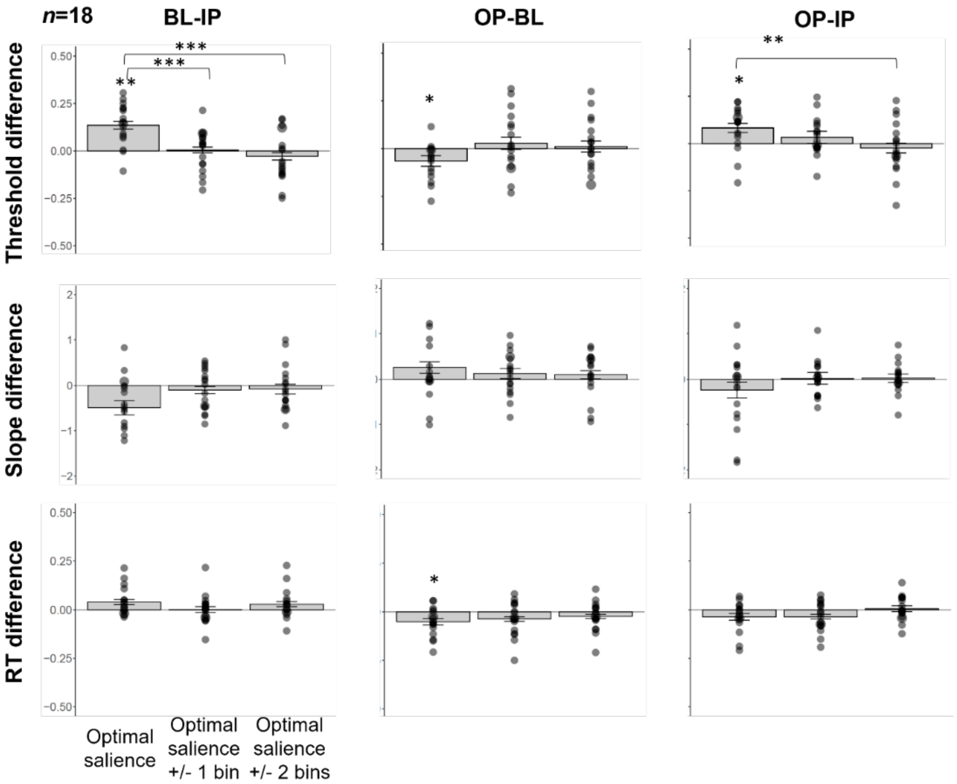

Figure S2. Threshold, slope, and RT difference aligned to individual participants' optimal auditory salience.

Table S1. Results of overall statistical analysis.

		BL vs. IP	OP vs. BL	OP vs. IP
Threshold difference	Mean threshold difference at mid-salience (*SD*)	-0.009 (0.137)	0.014 (0.144)	0.004 (0.172)
	t-test against zero for threshold difference at mid salience	$t(17) = -0.292$, $p = 0.774$	$t(17) = 0.400$, $p = 0.694$	$t(17) = 0.103$, $p = 0.919$
	F-test for the main effect of salience on threshold difference	$F(4,68) = 0.645$, $p = 0.632$, $\eta^2_G = 0.017$<br><br>Intercept: $F(1,17) = 0.992$, p $= 0.333$, $\eta^2_G = 0.031$	$F(4,68) = 1.598$, $p = 0.185$, $\eta^2_G = 0.051$<br><br>Intercept: $F(1,17) = 0.005$, p$=0.943$, $\eta^2_G < 0.001$	$F(4,68) = 1.044$, $p = 0.391$, $\eta^2_G = 0.049$<br><br>Intercept: $F(1,17) = 2.903$, p $= 0.107$, $\eta^2_G = 0.027$
Slope difference	Mean slope difference at mid-salience (*SD*)	-0.081 (0.563)	0.085 (0.714)	0.005 (0.511)
	t-test against zero for slope difference at mid salience	$t(17) = 0.608$, $p = 0.551$	$t(17) = 0.508$, $p = 0.618$	$t(17) = 0.038$, $p = 0.970$
	F-test for the main effect of salience on slope difference	$F(4,68) = 0.696$, $p = 0.598$, $\eta^2_G = 0.021$<br><br>Intercept: $F(1,17)=4.818$, $p = 0.042$, $\eta^2_G = 0.119$	$F(4,68) = 0.171$, $p = 0.953$, $\eta^2_G = 0.004$<br><br>Intercept: $F(1,17)=0.777$, $p = 0.390$, $\eta^2_G = 0.026$	$F(4,68) = 0.808$, $p = 0.524$, $\eta^2_G = 0.040$<br><br>Intercept: $F(1,17)=7.618$, $p = 0.013$, $\eta^2_G = 0.052$
RT difference	Mean RT difference at mid-salience (*SD*)	0.015 (0.066)	-0.029 (0.080)	-0.014 (0.073)
	t-test against zero for RT difference at mid salience	$t(17) = 0.999$, $p = 0.332$	$t(17) = 1.549$, $p = 0.140$	$t(17) = 0.804$, $p = 0.433$
	F-test for the main effect of salience on RT difference	$F(4,68) = 1.183$, $p = 0.326$, $\eta^2_G = 0.036$<br><br>Intercept: $F(1,17) = 1.102$, $p = 0.308$, $\eta^2_G = 0.029$	$F(4,68) = 4.856$, $p = 0.002$, $\eta^2_G = 0.082$<br><br>Intercept: $F(1,17) = 5.028$, $p = 0.039$, $\eta^2_G = 0.169$	$F(4,68) = 1.368$, $p = 0.254$, $\eta^2_G = 0.049$<br><br>Intercept: $F(1,17) = 5.702$, $p = 0.029$, $\eta^2_G = 0.106$

Table S2. Results of statistical analysis based on data aligned by optimal auditory salience.

		BL vs. IP	OP vs. BL	OP vs. IP
Threshold difference	Mean threshold difference at optimal salience (*SD*)	0.135 (0.106)	-0.064 (0.91)	0.083 (0.114)
	t-test against zero for threshold difference at optimal salience	$t(17) = 5.392$, $p < 0.002$	$t(17) = -3.002$, $p = 0.024$	$t(17) = 3.077$, $p = 0.021$
	F-test for the main effect of salience on threshold difference	$F(2,34) = 22.157$, $p < 0.001$, $\eta^2_G = 0.281$ * *Posthoc analysis: *p*s < 0.001 (Optimal vs. bin 1 & Optimal vs. bin 2)<br><br>Intercept: $F(1,17) = 2.640$, p =0.123, $\eta^2_G = 0.098$	$F(2,34) = 2.777$, $p = 0.076$, $\eta^2_G = 0.091$<br><br>Intercept: $F(1,17) = 0.197$, p=0.663, $\eta^2_G = 0.004$	$F(2,34) = 3.774$, $p = 0.033$, $\eta^2_G = 0.127$ * *Posthoc analysis: $p = 0.009$ (Optimal vs. bin 2)<br><br>Intercept: $F(1,17) = 3.747$, p =0.080, $\eta^2_G = 0.066$
Slope difference	Mean slope difference at optimal salience (*SD*)	-0.497 (0.826)	0.259 (0.797)	-0.239 (0.793)
	t-test against zero for slope difference at optimal salience	$t(17) = -2.555$, $p = 0.062$	$t(17) = 1.376$, $p = 0.654$	$t(17) = -1.278$, $p = 0.405$
	F-test for the main effect of salience on slope difference	$F(2,34) = 3.858$, $p = 0.031$, $\eta^2_G = 0.092$ * *Posthoc analysis: FDR-adjusted *p*s > 0.098<br><br>Intercept: $F(1,17)=4.506$, $p = 0.049$, $\eta^2_G = 0.128$	$F(2,34) = 0.620$, $p = 0.544$, $\eta^2_G = 0.014$<br><br>Intercept: $F(1,17) = 2.059$, $p = 0.169$, $\eta^2_G = 0.069$	$F(2,34) = 1.159$, $p = 0.326$, $\eta^2_G = 0.050$<br><br>Intercept: $F(1,17) = 1.189$, $p = 0.291$, $\eta^2_G = 0.016$
RT difference	Mean RT difference at optimal salience (*SD*)	0.040 (0.070)	-0.051 (0.070)	-0.036 (0.073)
	t-test against zero for RT difference at optimal salience	$t(17) = 2.418$, $p = 0.081$	$t(17) = -3.068$, $p = 0.021$	$t(17) = -2.100$, $p = 0.081$
	F-test for the main effect of salience on RT difference	$F(2,34) = 2.306$, $p = 0.115$, $\eta^2_G = 0.050$<br><br>Intercept: $F(1,17) = 3.009$, $p = 0.101$, $\eta^2_G = 0.098$	$F(2,34) = 1.050$, $p = 0.361$, $\eta^2_G = 0.023$<br><br>Intercept: $F(1,17) = 6.868$, $p = 0.018$, $\eta^2_G = 0.202$	$F(2,34) = 2.551$, $p = 0.093$, $\eta^2_G = 0.075$<br><br>Intercept: $F(1,17) = 4.021$, $p = 0.061$, $\eta^2_G = 0.098$
